# A Case of Uterine Lymphangioleiomyomatosis Complicated by Tuberous Sclerosis Complex

**DOI:** 10.1155/2022/2893975

**Published:** 2022-12-13

**Authors:** Kaori Yamada, Yukio Yamanishi, Junichi Aratake, Nanayo Sasagasako, Yoshihide Inayama, Rei Gou, Atsuko Kawamura, Megumi Yamanishi, Kenzo Kosaka

**Affiliations:** Department of Obstetrics and Gynecology, Shizuoka Prefectural General Hospital, Shizuoka, Japan

## Abstract

Lymphangioleiomyomatosis (LAM) is one of the presentations of perivascular epithelioid cell neoplasm that is frequently complicated by tuberous sclerosis complex (TSC). Here, we report an uncommon case of uterine LAM treated with everolimus, which is a mechanistic target of rapamycin (mTOR) inhibitor. A 42-year-old female patient (gravida 0) with a history of TSC presented with abdominal pain. Pelvic magnetic resonance imaging showed multiple masses in the uterine myometrium, suggesting tumors that may contain internal hemorrhagic components. The lesions were suspected as the root cause of her symptoms. After everolimus was administered for a previously diagnosed renal angiolipoma, her uterine tumors temporarily decreased in size. Subsequently, laparoscopic hysterectomy and bilateral salpingectomy were performed since she could not tolerate everolimus for a long period due to the medication's side effects. Furthermore, the patient was diagnosed with LAM through histopathological examination after surgical resection. Therefore, it is advisable to suspect and investigate uterine LAM when a patient with a history of TSC presents with irregular genital bleeding or abdominal pain. Moreover, mTOR inhibitors may be a treatment option, in addition to surgery, in cases of uterine LAM exacerbation.

## 1. Introduction

Perivascular epithelioid cell tumors (PEComas) are tumors composed of cells depicting perivascular epithelioid cell differentiation [1]. These cells have clear to eosinophilic cytoplasm and exhibit positive staining for smooth muscle and melanocytic markers. They are classified as follows: angiomyolipomas, clear cell “sugar” tumors, clear cell myomelanocytic tumors of the falciform ligament/ligament teres, and lymphangioleiomyomatosis (LAM). PEComas of the gynecological tract are rare, with uterine PEComas accounting for most of these cases [2]. Irregular genital bleeding and lower abdominal pain are common symptoms of uterine PEComas. Although most cases are benign, some cases of malignant PEComas metastasize or recur [3].

LAM is one of the presentations of PEComas that occurs in women of reproductive age. It is generally found in the lungs and lymph nodes but can also occur in the pelvic organs, including the kidneys and uterus. To the best of our knowledge, uterine LAM is a rare disease, and only less than 100 cases have been reported [4]. LAM is caused by mutations in the *TSC*1 and *TSC*2 genes, resulting in the inactivation of these genes. Some cases are solitary (sporadic LAM) [4]. However, many cases of LAM have been reported to be complicated by tuberous sclerosis (TSC; TSC-LAM).

Here, we report an unusual case of LAM complicated by TSC.

## 2. Case Presentation

**Figure 1:**
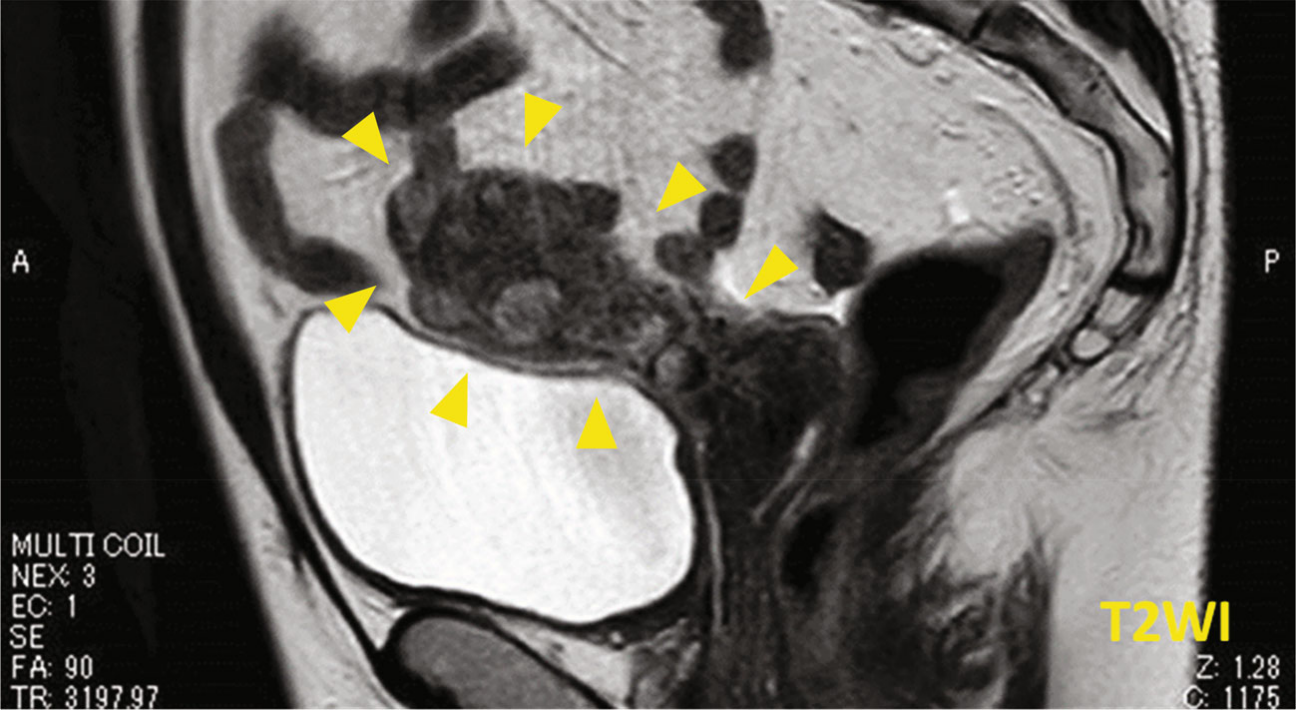
Magnetic resonance imaging (MRI) revealed multiple masses extending from the posterior wall of the uterus to the basal layer of the myometrium.

**Figure 2:**
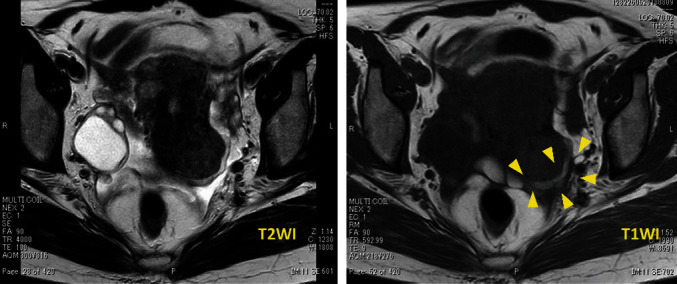
*T*1-weighted and *T*2-weighted images revealed a higher intensity, suggesting the tumor containing internal hemorrhagic components.

**Figure 3:**
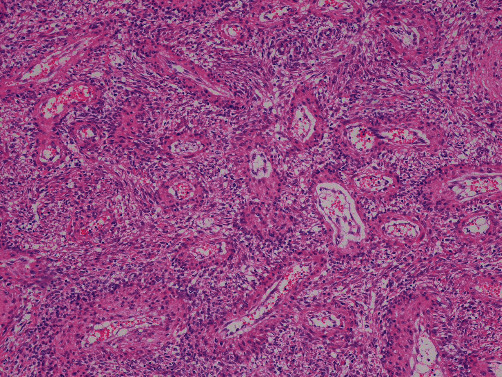
Numerous vessels and areas of a perivascular arrangement were observed (Hematoxylin and eosin (HE) stain　×100).

**Figure 4:**
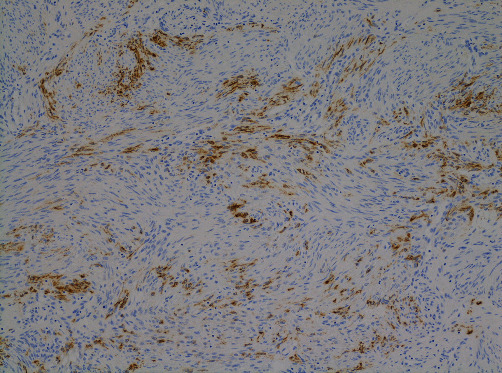
Immunohistochemistry of the tumor cells showing positive staining for HMB-45 (100×).

A 42-year-old female patient (gravida 0) presented with a major complaint of longstanding abdominal pain lasting over a year. She had a pertinent history of TSC and antiphospholipid antibody syndrome. She was diagnosed clinically for TSC, without any genetic test (*TSC*1 and *TSC*2). She also had multiple LAM lesions in the lungs accompanied by other lesions associated with TSC, such as renal-angiomyolipoma and angiofibroma. She was followed up regularly using respiratory medicine, neurology, and urology with imaging studies. The lung lesions particularly required special attention, despite the stable disease status. Magnetic resonance imaging (MRI) showed multiple masses extending from the basal layer of the myometrium to the posterior wall of the uterus (Figure 1). *T*1- and *T*2-weighted images revealed areas of higher intensity, suggesting tumors that could contain internal hemorrhagic components (Figure 2). The endometrium was 5 mm. Diffusion restriction was not observed, and the apparent diffusion coefficient values were not lower than those for normal myometrium. Differential diagnoses included uterine adenomyosis and endometriotic lesions rather than malignant tumors, such as uterine sarcoma, because the uterine masses were diffusely extended. Subsequently, everolimus (10 mg/day), which is a mechanistic target of rapamycin (mTOR) inhibitor, was initiated for concomitant renal angiolipoma but was discontinued approximately after a month, as the patient contracted stomatitis as a side effect. The patient's abdominal pain improved while she was being treated with everolimus. However, her abdominal pain worsened the following year, which was accompanied by irregular genital bleeding. Pelvic MRI showed that the lesions, which had previously decreased in size, were enlarged. Uterine lesions caused by TSC, along with endometrial lesions, were suspected as the cause of her symptoms. After careful counseling, laparoscopic hysterectomy and bilateral salpingectomy were performed with the patient's strong request. On gross examination, a myomatous nodule, which was 1.5 cm in diameter, was observed on the posterior wall of the uterus. In addition, diffuse, patchy, and partially nodular lesions were observed in the myometrium. Microscopically, indistinct borders and bright areas of infiltrative growth resembling endometrial stromal sarcomas were observed in the myometrium. Spindle-shaped cells with clear or eosinophilic granular cytoplasm proliferated with slit-like spaces lined by endothelial cells. Invasion of the endometrium and concurrent adenomyosis were also observed. Numerous vessels and areas infiltrated by perivascular arrangements were observed (Figure 3). Neither mitotic nor necrotic activity was found. Immunohistochemically, the tumor was positive for Human melanin black 45 (HMB-45), *α*- smooth muscle actin (*α*-SMA), and desmin and partially positive for Melan-A (Figure 4). Based on these pathological findings, the patient was diagnosed with LAM. Notably, 4 years after surgery, the patient had no evidence of disease recurrence.

## 3. Discussion

Approximately one-third of women with TSC have comorbid LAM [5, 6]. Therefore, when a patient with a history of TSC presents with irregular genital bleeding and abdominal pain, the uterine lesions should be investigated, considering LAM or PEComa.

The characteristic imaging findings of LAM or PEComa have not been firmly established. According to reports, ultrasound examinations generally tend to show heterogeneous echogenicity and abundant vessels [7, 8], whereas MRI findings generally appear as low- and high-signal intensities on *T*1- and *T*2-weighted images, respectively. However, these findings vary [9]. In this case, a diffuse, poorly demarcated lesion was found in the myometrium and required differentiation from cystic adenomyosis and pelvic endometriosis. Although the final diagnosis depends on histopathological examination after surgical resection, knowledge of the characteristic findings of LAM or PEComa could help improve the diagnosis. Therefore, MRI can be recommended as the first diagnostic method if LAM is highly suspected. Tumor biopsy can also be considered, but its efficacy has not been firmly established. Some cases of preoperative biopsies have been reported [3]. However, the amount of tissue obtained from biopsies is minute and does not easily lead to a definitive diagnosis.

The standard treatment for uterine LAM is complete surgical resection. Recently, mTOR inhibitors were reported to be useful for treating malignant or metastatic PEComas [10–12]. In patients with TSC, mutations in the *TSC*1 and *TSC*2 genes cause excessive mTOR activation due to the dysregulation of the PI3K/AKT/mTOR signaling pathway. mTOR inhibitors are considered effective in various diseases, such as renal angiomyolipoma and epilepsy associated with TSC [13].

Microscopically, in patients with uterine LAM associated with TSC, tongue-like growth patterns and HMB-45-positive epithelioid cells are more prominent histological findings compared with sporadic LAM. Uterine LAM is present in approximately 90% of patients with LAM in the lungs [14]. Furthermore, uterine LAM may be present even in the absence of overt symptoms in patients with TSC; therefore, active monitoring of lesions may be useful for early diagnosis and treatment.

In this case, everolimus, which is an mTOR inhibitor, was used to treat the patient's renal angiomyolipoma. Because the treatment period was brief, no assessment of its efficacy could be performed. However, the treatment slightly diminished the uterine lesions, and the abdominal pain was resolved. Moreover, if the treatment had been administered longer, the lesions could have been further controlled.

Patients with TSC have higher perioperative risks, such as renal dysfunction, renal hypertension, epilepsy, pneumothorax, and pulmonary hypertension, when considering surgery under general anesthesia [15]. In such cases, conservative treatment with mTOR inhibitors may be an option if malignancy or metastasis is not suspected. However, only a few reports of cases of uterine LAM exist, without malignant and metastatic cases, which were treated with mTOR inhibitors; therefore, further studies are required.

## 4. Conclusions

When a patient with a history of TSC presents with irregular genital bleeding or abdominal pain, it is advisable to suspect and investigate uterine LAM. Therefore, in addition to surgery, mTOR inhibitors may be a treatment option, particularly in cases of uterine LAM exacerbation.

## Data Availability

Data sharing is not applicable to this article as no new data were created or analyzed in this study.
